# Correcting On-the-Go Field Measurement–Coordinate Mismatch by Minimizing Nearest Neighbor Difference

**DOI:** 10.3390/s22041496

**Published:** 2022-02-15

**Authors:** Alfonso González Jiménez, Yakov Pachepsky, José Luis Gómez Flores, Mario Ramos Rodríguez, Karl Vanderlinden

**Affiliations:** 1 IFAPA Centro Alameda del Obispo, 14004 Córdoba, Spain; trabajoaj303@gmail.com (A.G.J.); josel.gomez@juntadeandalucia.es (J.L.G.F.); mario.ramos.r@juntadeandalucia.es (M.R.R.); 2 USDA-ARS Environmental Microbial and Food Safety Laboratory, Beltsville, MD 20705, USA; yakov.pachepsky@usda.gov

**Keywords:** coordinate translation, electromagnetic induction, measurement-coordinate mismatch, nearest neighbor, on-the-go field measurements, time lag

## Abstract

Many current precision agriculture applications involve on-the-go field measurements of soil and plant properties that require accurate georeferencing. Specific equipment configuration characteristics or data transmission, reception, or logging delays may cause a mismatch between the logged data and the GPS coordinates because of time and position lags that occur during data acquisition. We propose a simple coordinate translation along the measurement tracks to correct for such positional inaccuracies, based on the local travel speed and time lag, which is estimated by minimizing the average ln-transformed absolute difference with the nearest neighbors. The correction method is evaluated using electromagnetic induction soil-sensor data for different spatial measurement layouts and densities and by comparing variograms for raw and modified coordinates. Time lags of 1 s are shown to propagate into the spatial correlation structure up to lag distances of 10 m. The correction method performs best when repeated measurements in opposite driving directions are used and worst when measurements along parallel driving tracks are only repeated at the headland turns. In the latter case, the performance of the method is further improved by limiting the search neighborhood to adjacent measurement tracks. The proposed coordinate correction method is useful for improving the positional accuracy in a wide range of soil- and plant-sensing applications, without the need to grid the data first.

## 1. Introduction

Motorized on-the-go field measurements involve digital data flows from different devices, including soil sensors (e.g., electromagnetic induction (EMI) sensors), grain yield sensors, and GPS receivers. The data provided by each device are then stored on a common platform such as a data logger or field computer with a specific timestamp. Depending on sensor type, cable lengths, communication and hardware configurations, and measurement platform design, delays can occur in the reception and storage of the data. Depending on local measurement speed, the resulting time lag propagates into a position lag as the measurements are linked with the wrong GPS coordinates. This leads to “sawtooth” patterns when the data are interpolated, particularly when measurements are performed along adjacent parallel tracks with opposite driving directions.

These and other data accuracy issues have received considerable attention in the context of anomaly detection in archaeology [1] and particularly crop yield mapping [2,3,4,5], where time lags occur between the cutting of the crop and the measurement by the grain flow sensor in the harvester, and a constant horizontal offset exists depending on the position of the GPS antenna on the harvester. In [6], the authors proposed a computationally efficient method to estimate the time lag from image processing with the phase correlation method, implemented in the yield editor tool presented by [3]. This method requires the data to be interpolated first and mapped on a raster image. The importance of positional accuracy was also recognized in the context of EMI surveys in soil-mapping applications (e.g., [7]). In this context, it is common for practical reasons to place the GPS antenna on the towing vehicle and not above the sensor, resulting in a constant horizontal offset between the GPS and the measurement position. Such cases have been addressed [8,9] through comparisons and evaluations of several corrections, based on constant translations, for a constant offset between an EMI sensor and the GPS position. The problem also differs substantially from the coordinate corrections proposed for autonomous vehicle applications where fast online coordinate corrections are required for updating real-time position estimates [10].

To the best of our knowledge, we are unaware of any coordinate correction method that accounts for an unknown time lag in on-the-go field sensing applications. Therefore, the objective of this work is to develop a simple method to correct coordinates of on-the-go field measurements by minimizing the average absolute difference between nearest neighbors. The performance of the method is evaluated using soil EMI measurements. The correction method and the measurement layouts used are presented in Section 2, and Section 3 is dedicated to the evaluation of the correction method for different spatial measurement layouts and recommendations for optimal performance. The conclusions are presented in Section 4.

## 2. Materials and Methods

### 2.1. Correction Method

**Figure 1:**
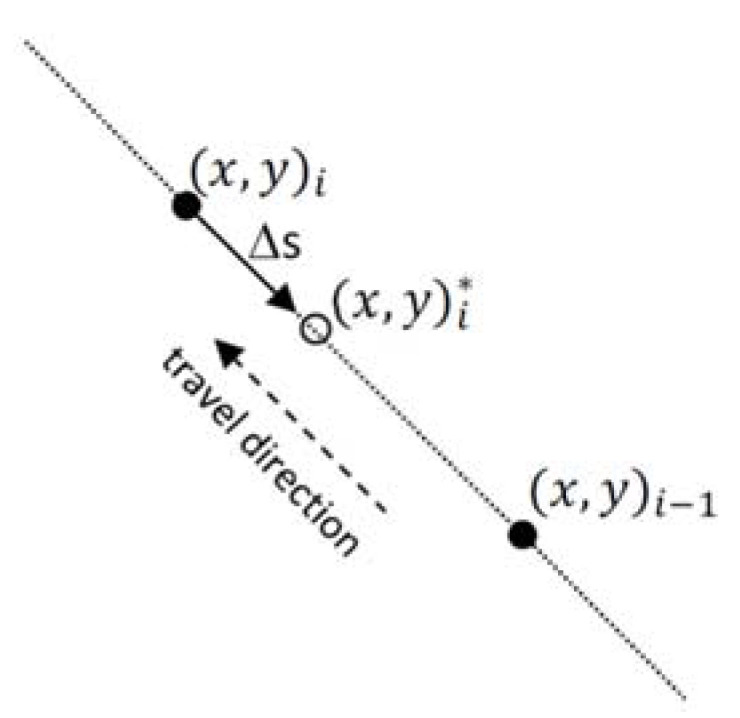
Schematic representation of the translation, Δ*s*, used to correct the spatial coordinates. The filled circles represent two consecutive original measurement locations, ${\left(x,y\right)}_{i-1}$, and ${\left(x,y\right)}_{i}$, while the empty circle represents the corrected location, ${\left(x,y\right)}_{i}^{*}$, of the latter measurement.

The correction is based on a coordinate translation, Δ*s* (m), of the measurement locations along the track of travel and depending on the local speed, *v*_i_ (m s^−1^), and a measurement-configuration-specific unknown constant time lag, Δ*t* (s), as shown in Figure 1. The corrected coordinates, ${\left(x,y\right)}_{i}^{*}$ of the original measurement location ${\left(x,y\right)}_{i}$, are calculated according to

(1) $$x_{i}^{*}=x_{i}+\frac{x_{i-1}-x_{i}}{\sqrt{{\left(x_{i}-x_{i-1}\right)}^{2}+{\left(y_{i}-y_{i-1}\right)}^{2}}}v_{i}\Delta t$$

(2) $$y_{i}^{*}=y_{i}+\frac{y_{i-1}-y_{i}}{\sqrt{{\left(x_{i}-x_{i-1}\right)}^{2}+{\left(y_{i}-y_{i-1}\right)}^{2}}}v_{i}\Delta t.$$

The optimal value of Δ*t* is found by minimizing the average ln-transformed absolute difference,

(3) $$\bar{\ln\left|\Delta z\right|}=\frac{\sum_{i=1}^{n}\ln{\left|\Delta z\right|}_{i}}{n}$$

between measurements and their nearest neighbors, with *z* being the measured property and *n* being the number of data points. This is achieved by successively inserting Δ*t* values ranging from 0.1 to 2 s in Equations (1) and (2) to calculate the corresponding “corrected” coordinate sets. For each set of coordinates, $\bar{\ln\left|\Delta z\right|}$ is calculated and represented as a function of Δ*t*. Subsequently, a spline function with tension factor equal to two is fitted to the Δ*t*-$\bar{\ln\left|\Delta z\right|}$ data pairs, and the optimal Δ*t* is identified as the minimum of this spline function. The optimal Δ*t* is then used in Equations (1) and (2) to calculate the corrected coordinates.

A local square search neighborhood was used to optimize the nearest neighbor search and minimize computation time. The size of the square depends on the spatial data density and configuration and is a user-defined input parameter for the search algorithm. The nearest neighbor search is either performed considering all the data points in the search neighborhood (search strategy 1 (S1)) or considering only data points from adjacent measurement tracks (search strategy 2 (S2)). The correction method was implemented in R [11].

### 2.2. Data Acquisition and Processing

We used apparent electrical conductivity (ECa) data, measured with a DUALEM-21S (DUALEM, Milton, ON, Canada) EMI sensor, to evaluate the proposed correction method (Equations (1)–(3)). Details of the use of EMI sensors in soil studies can be found in [12]. The measurements were made on 9 September 2020, between 10:00 a.m. and 1:00 p.m. on a recently laser-leveled 12.5 ha field in the B-XII irrigation district (Lebrija, Seville) in southwest Spain. This area consists of reclaimed saline marshes characterized by expansive heavy clay soils and a shallow saline water table below the drainage system installed at approximately 1 m depth and with an average distance between the parallel 250 m long drainage pipes of 5 m. Further details of the study area and its soil can be found in [13].

**Figure 2:**
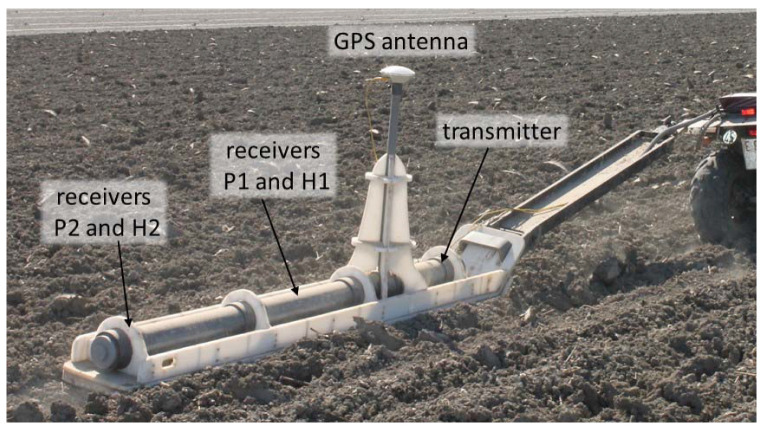
PVC sled used to measure apparent electrical conductivity with a DUALEM-21S electromagnetic induction sensor, with the positions of the GPS antenna, the transmitter and the four receiver coils indicated.

The EMI sensor was housed in a customized polyvinyl chloride (PVC) sled (Figure 2) at a height of 0.105 m above the soil surface and towed by an all-terrain vehicle (ATV) which was equipped with a mesa^3^ field computer (Juniper Systems, Logan, UT, USA) for data collection and storage. A real-time kinematic differential global positioning system (Trimble, Sunnyvale, CA, USA) was used for georeferencing the EMI measurements and measurement of terrain elevation. To provide more stability to the sled and to prevent overturning, it was connected to the ATV using a rigid articulated arm (Figure 2).

The DUALEM-21S contains four receiver coils in perpendicular (P) and horizontal co-planar (H) configurations at 1.1 (P1), 1 (H1), 2.1 (P2), and 2 m (H2) from the transmitter coil (Figure 2), providing theoretical depths of exploration (DOE) of approximately 0.5, 1.5, 1.0, and 3.0 m, respectively. Detailed information on the DUALEM-21S sensor can be found in [14]. The GPS antenna was located on the PVC sled at a height of 1.5 m in the center of the H1 coil configuration, producing a constant offset between the center of the four coil configurations and the GPS antenna of 0.05, 0, 0.55, and 0.50 m, respectively. Further details of a similar set-up are available in [15].

During the field measurement, geographical RTK-DGPS coordinates were logged once per second, while the four DUALEM-21S signals were measured twice per second. The 1 (P1 and H1) and 2 m (P2 and H2) signals were logged with different timestamps. Driving speed was also recorded. Geographical coordinates were converted to the UTM system in order to perform further data processing in a Cartesian system in which Euclidean distance can be used. The UTM-transformed RTK-DGPS coordinates were then interpolated according to the timestamps of the H and P signals provided by the sensor clock, which had a resolution of 0.01 s. Further processing involved the detection and removal of extreme values and measurements made at speeds < 0.5 km/h from the dataset. The H1 signal was used for evaluation purposes. Its DOE of 1.5 m usually provided the most stable and representative measurements for the soil profile. For demonstration purposes, the four signals were used. In accordance with the dataset used hereinafter to evaluate the performance of the proposed correction method, a range of Δ*t* values from 0.1 to 2 s was adopted. The length of the side of the square search neighborhood ranged from 2 m (S2) to 10 m (S1).

### 2.3. Spatial Measurement Layout

**Figure 3:**
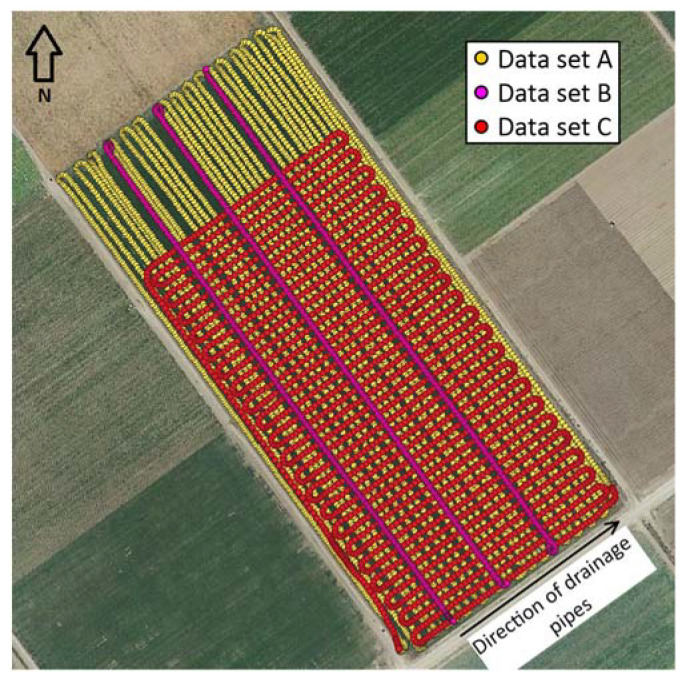
Spatial layouts of the ECa measurements used for the evaluation of the correction method. Data set A: measurements performed with driving direction perpendicular to the drainage pipes; data set B: measurements along three lines duplicating the corresponding lines in data set A, but with opposite driving directions; and data set C: measurements performed in the direction of the drainage pipes.

Apparent electrical conductivity measurements were made in the direction perpendicular to the drainage pipes at an average speed of 9 km/h and a density of 0.19 points/m^2^ (Table 1 and Figure 3; data set A). In addition, three measurement lines were duplicated in opposite driving directions, as shown in Figure 3 (data set B). The descriptive statistics for data set B are not included in Table 1 since this data set was only used for evaluation purposes in combination with data sets A and C. Subsequently, measurements were made in the direction of the drainage pipes at an average speed of 14 km/h, yielding a density of 0.11 points/m^2^ (Table 1 and Figure 3; data set C). The correction method was evaluated using different combinations of these data sets, resulting in different spatial measurement layouts and data densities for the H1 signal, as shown in Table 1.

Overall, the average ECa and coefficient of variation (CV) increased and decreased with depth, respectively (Table 1). Except for data set C, similar CVs were obtained for each combination of the data sets. The average ECa was slightly higher for data set C than for the other data set combinations since the northwest part of the field with smaller ECa values could not be measured for this measurement layout due to technical issues related to the measurement equipment and was therefore not included in data set C (Figure 3).

## 3. Results and Discussion

### 3.1. Effect of Spatial Measurement Layout on the Nearest Neighbor Differences

**Figure 4:**
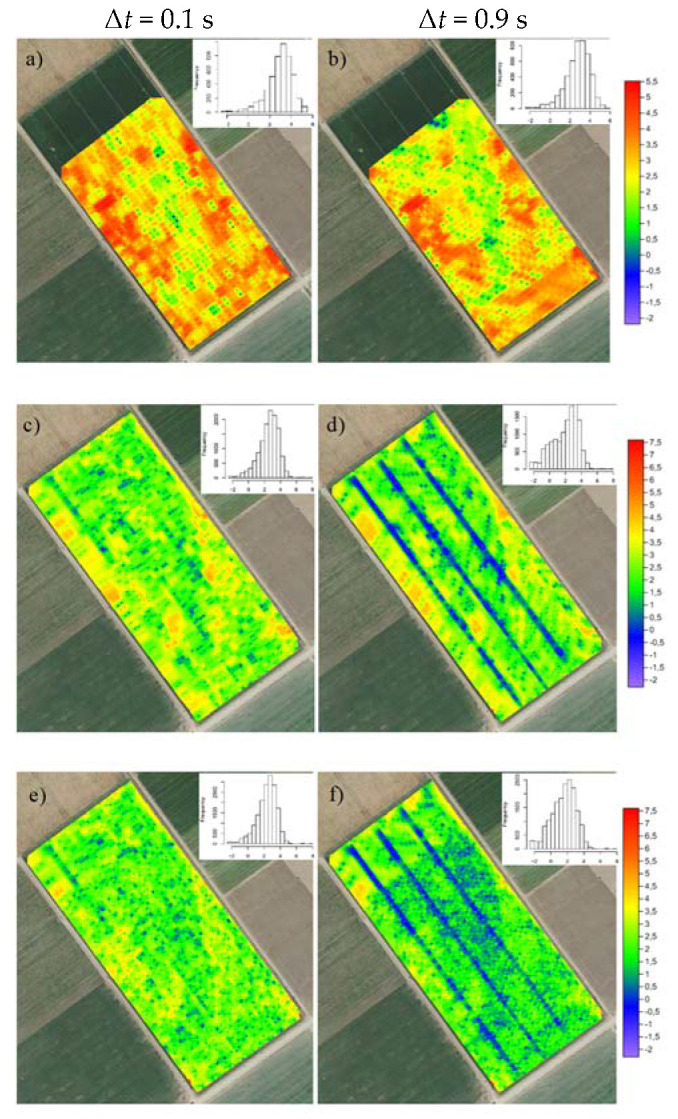
Maps and histograms of ln-transformed absolute nearest neighbor difference distribution (ln|Δ*z*|) of H1 ECa. (**a**,**c**,**e**), non-optimized (Δ*t* = 0.1 s) and (**b**,**d**,**f**) optimized (Δ*t* = 0.9 s) time lags (Δ*t*) for data set combinations C (**a**,**b**), A + B (**c**,**d**), and A + B + C (**e**,**f**).

Figure 4 shows the spatial distribution and the histograms of the ln-transformed absolute nearest neighbor difference distribution of ECa (ln|Δz|) for the H1 signal using the S2 search method and data set combinations C (Figure 4a,b), A + B (Figure 4c,d) and A + B + C (Figure 4e,f) for non-optimized (Δ*t* = 0.1 s) and optimized (Δ*t* = 0.9 s) time lags.

As expected, ln|Δz| was largest for the non-optimized Δ*t* (Figure 4a,c,e) and in areas with sharp transitions between large and small ECa values. The values of ln|Δ*z*| became particularly small when spatially dense ECa data sets were used, as can be seen in Figure 4d for dataset A + B along the lines where measurements were made in opposite driving directions. When combining all the data (A + B + C), smaller ln|Δz| values were observed across the entire field, as shown in Figure 4e,f. The maps and histograms in Figure 4 show that ln|Δ*z*| followed a near-normal distribution, allowing robust estimation of $\bar{\ln\left|\Delta z\right|}$ (Equation (3)) to find the optimal correction.

### 3.2. Comparison of Optimization Methods S1 and S2

**Figure 5:**
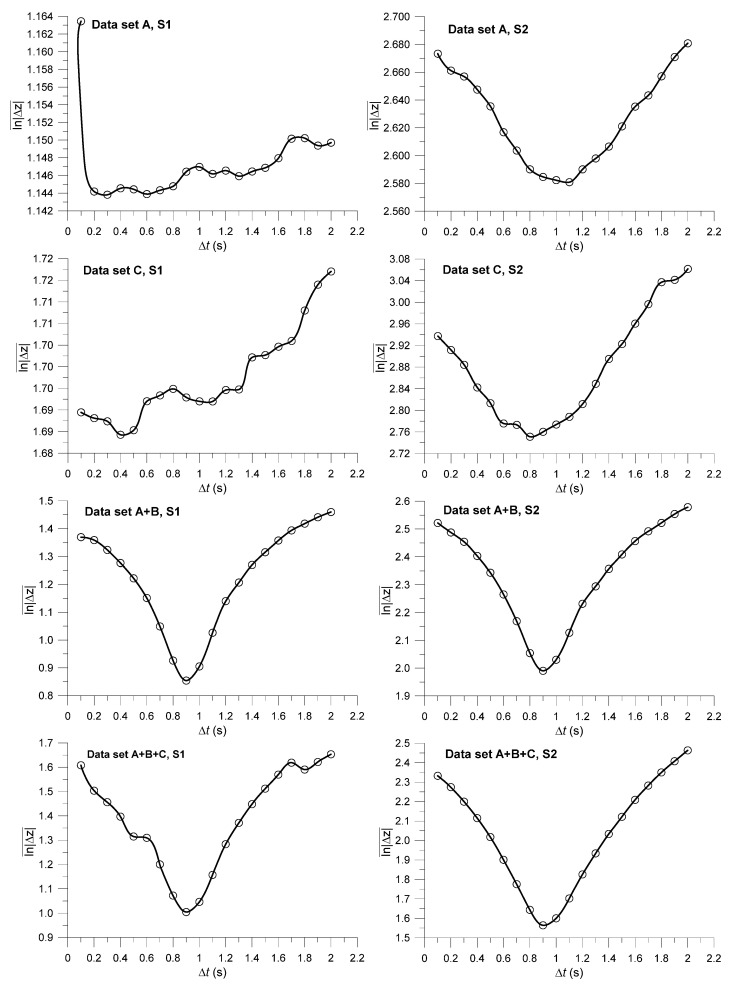
Relationships between $\bar{\ln\left|\Delta z\right|}$ and Δ*t* using search methods S1 (**left**) and S2 (**right**) for H1 ECa and 4 different data set combinations. The fitted line is a spline function with a tension factor equal to two.

Figure 5 shows $\;\bar{\ln\left|\Delta z\right|}$, calculated using Equations (1)–(3) with the H1 ECa signal for Δ*t* ranging from 0.1 to 2 s. The search method S2 is, in general, capable of identifying a minimum of $\bar{\ln\left|\Delta z\right|}$, as opposed to S1, because S2 excludes nearest neighbors from the same measurement tracks. Because the correction is based on a linear translation (Equations (1) and (2), Figure 1), $\bar{\ln\left|\Delta z\right|}$ remains constant for different Δ*t* if the nearest neighbors are located on the same measurement track. As a result, S1 is less robust and provides only suitable results if overlapping measurements are available (e.g., data sets A + B and A + B + C).

### 3.3. Inferring the Optimal Time Lag

The minimum $\bar{\ln\left|\Delta z\right|}$ and the corresponding optimal Δ*t* were identified as the minimum of the spline functions, as shown in Figure 5. Table 2 shows the optimized Δ*t* and the corresponding average spatial offset 〈Δ*s*〉, calculated using the average speed 〈*v*〉 shown in Table 1, for the four different signals, data configurations, and nearest neighbor search methods (S1 and S2). The obtained time lags for S1 and S2 were similar when overlapping measurements were available (A + B and A + B + C). If no overlapping measurements were available (A and C), then only S2 provided Δ*t* values similar to those obtained for A + B and A + B + C. 〈Δ*s*〉 depended strongly on the driving speed during measurement. For the 1 and 2 m coil combinations, 〈Δ*s*〉 ranged from 2.0 to 3.3 m and 2.6 to 4.0 m, respectively.

### 3.4. Effect of the Coordinate Correction on the Spatial Correlation Structure

**Figure 6:**
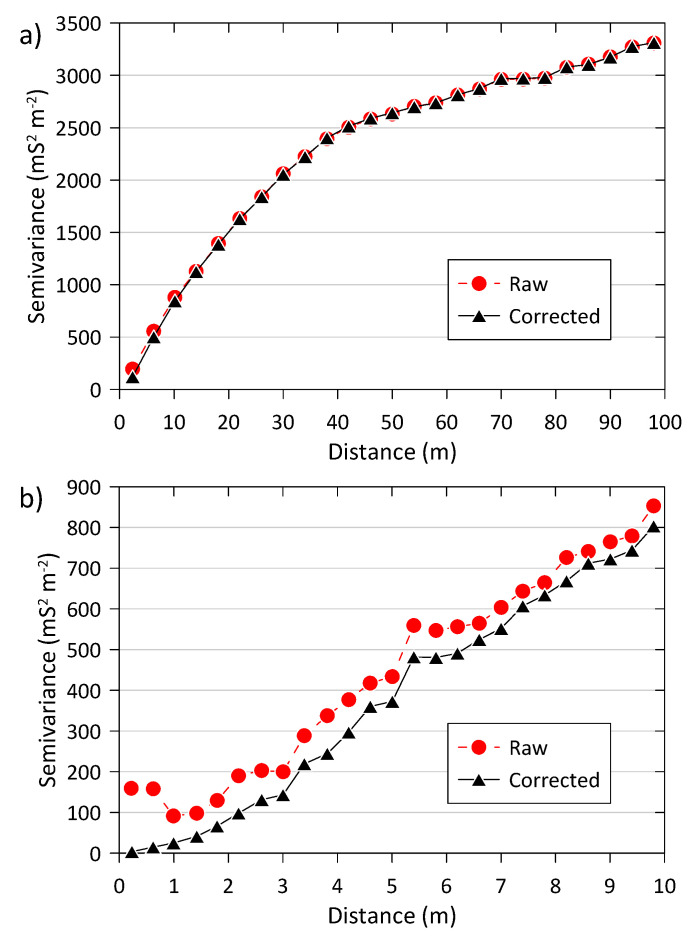
Variograms for the raw and corrected coordinates of the H1 ECa data using data set A + B + C. (**a**) Coarse-scale, up to a maximum lag distance of 100 m; (**b**) fine-scale, up to a maximum lag distance of 10 m.

Figure 6a shows the variograms for the raw and corrected coordinates of the H1 ECa data (data set A + B + C) at the coarse scale, up to a lag distance of 100 m. The raw and corrected variograms were almost identical, except for the first three lags (up to 10 m) where the semivariance was larger for the raw data. When zooming into the first lags (Figure 6b), it can be seen how the semivariance of the raw coordinates increased toward the origin for the smallest lags (roughly <1 m) compared to that of the corrected coordinates, which showed a smoothly varying spatial correlation structure near the origin. The effect of a ~1 s time lag propagated into the spatial correlation structure up to lags of 10 m in this case.

**Figure 7:**
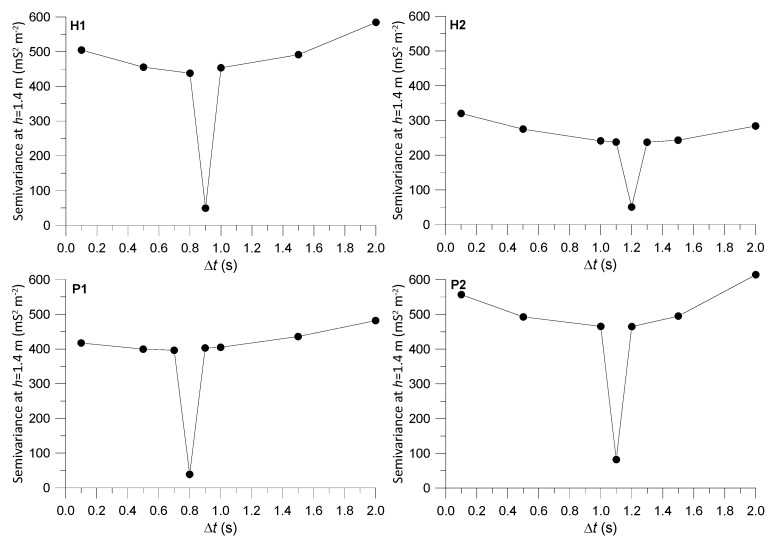
Semivariance at the first lag distance (*h* = 1.4 m) for different Δ*t* and ECa signals (H1, H2, P1 and P2) using data set A + B + C.

This smoothing effect of the coordinate correction near the origin of the variograms is also illustrated in Figure 7, showing semivariance at the first lag distance (*h* = 1.4 m), a proxy for the nugget effect, for different Δ*t* and ECa signals using data set A + B + C. A clear minimum was observed in the semivariance values near the optimal Δ*t*. The existence of such minima confirmed that the correction also optimizes the fine-scale spatial correlation structure by minimizing short-range variability and resulting in smoother interpolated ECa maps.

### 3.5. Corrected ECa Maps

**Figure 8:**
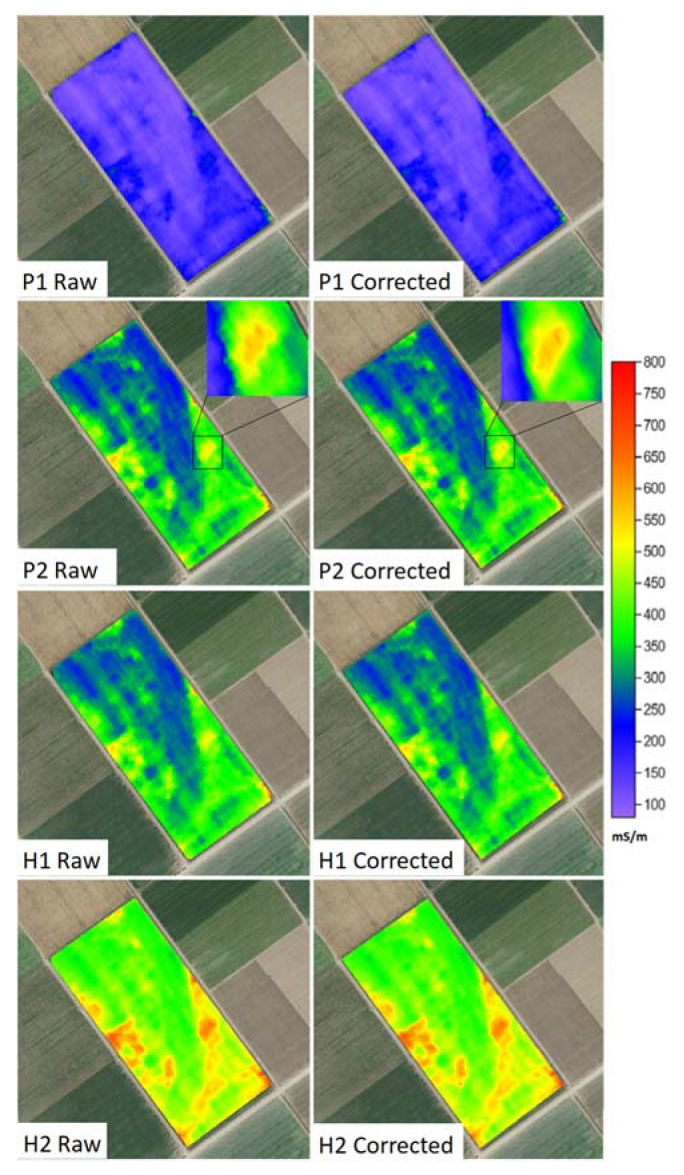
ECa maps for the four signals with raw and corrected coordinates. The inset for the P2 signal shows in detail how the sawtooth pattern disappears for the corrected coordinates.

The correction of the coordinates for the different ECa signals was based on data set A + B + C, which included all the available data, and the S2 nearest neighbor search method. Figure 8 shows the maps for the four ECa signals with raw and corrected coordinates. At the coarse (field) scale, spatial patterns were similar for the raw and corrected maps. Yet, at the fine scale (inset for P2), the sawtooth pattern in the raw data map disappeared after the coordinate correction. This example shows that the coordinate correction is particularly relevant for applications where fine-scale positional accuracy is important. Spatial consistency of the ECa signals becomes very important when the data are further processed using, for example, inversion software for estimating depth-specific soil EC (e.g., [16,17]). Other examples include comparison of ECa with specific soil properties measured at the point scale or comparison with remote or airborne imagery.

## 4. Conclusions

We developed a correction method for the coordinates of delayed on-the-go field measurements using a linear translation Δ*s* along the driving track, which was optimized by searching for a recording time lag that minimizes the average ln-transformed absolute difference with the nearest neighbor. The method was validated using the four ECa signals provided by a DUALEM-21S. Different spatial data layouts and two different nearest neighbor search algorithms were compared. Overall, the best results were obtained with both search algorithms if data density was high and partially overlapping measurement tracks were available. The best-case scenario was the one with overlapping ECa measurements obtained in opposite driving directions. When correcting legacy data, where it is impossible to obtain additional measurements, the S2 search method should be used, excluding measurements on the same measurement track from the nearest neighbor search so that only points on adjacent measurement tracks qualify as nearest neighbors.

## Figures and Tables

**Table 1:** Characteristics of the different combined ECa data sets and descriptive statistics for the different EMI signals. The total number of measurements (*n*), average speed (〈*v*〉, km/h), the average spacing between measurement lines (*p*, m), spatial data density (*d*, points/m^2^) and average ECa (〈ECa〉, mS/m), and coefficient of variation (CV, %).

Combination of Data Sets	A	C	A + B	A + B + C
*n*	42,100	16,700	48,000	64,700
〈*v*〉	9.1	14.2	8.8	9.9
*p*	7.0	9.4	6.4	
*d*	0.19	0.11	0.22	0.30
〈ECa〉 P1	147.7	152.2	145.5	149.2
〈ECa〉 H1	347.9	357.4	344.2	350.8
〈ECa〉 P2	344.9	354.9	341.0	348.1
〈ECa〉 H2	463.3	474.6	459.9	466.8
CV P1	38.0	26.8	37.3	35.3
CV H1	23.7	18.5	23.3	22.7
CV P2	25.4	21.1	25.1	24.8
CV H2	16.2	13.5	16.0	15.9

**Table 2:** Optimized time lags (Δ*t*) and average spatial offsets 〈Δ*s*〉, for the four ECa signals, considering different combined datasets and nearest neighbor search methods (S1 and S2).

	A		C		A + B		A + B + C	
	S1	S2	S1	S2	S1	S2	S1	S2
H1 Δ*t* (s)	0.25	1.02	0.42	0.85	0.91	0.92	0.92	0.92
P1 Δ*t* (s)	0.64	0.81	0.24	0.82	0.80	0.80	0.84	0.87
H2 Δ*t* (s)	0.46	1.31	1.80	1.04	1.29	1.30	1.28	1.26
P2 Δ*t* (s)	0.71	1.24	1.23	1.01	1.08	1.13	1.10	1.08
H1 〈Δ*s*〉 (m)	0.63	2.58	1.66	3.35	2.23	2.25	2.53	2.53
P1 〈Δ*s*〉 (m)	1.62	2.05	0.95	3.23	1.96	1.96	2.31	2.39
H2 〈Δ*s*〉 (m)	1.16	3.31	7.10	4.10	3.16	3.18	3.52	3.47
P2 〈Δ*s*〉 (m)	1.80	3.14	4.85	3.98	2.64	2.77	3.03	2.97

## Data Availability

Not applicable.
